# Neuroprotective Effect of Gui Zhi (*Ramulus Cinnamomi*) on Ma Huang- (*Herb Ephedra-*) Induced Toxicity in Rats Treated with a Ma Huang-Gui Zhi Herb Pair

**DOI:** 10.1155/2015/913461

**Published:** 2015-01-26

**Authors:** Fang-hao Zheng, Ping Wei, Hui-ling Huo, Xue-feng Xing, Fei-long Chen, Xiao-mei Tan, Jia-bo Luo

**Affiliations:** ^1^School of Chinese Medical Sciences, Southern Medical University, 510515 Guangzhou, China; ^2^Guangdong Province Key Laboratory of Chinese Medicine Pharmaceutics, Southern Medical University, 510515 Guangzhou, China

## Abstract

*Herb Ephedra* (Ma Huang in Chinese) and *Ramulus Cinnamomi* (Gui Zhi in Chinese) are traditional Chinese herbs, often used together to treat asthma, nose and lung congestion, and fever with anhidrosis. Due to the adverse effects of ephedrine, clinical use of Ma Huang is restricted. However, Gui Zhi extract has been reported to decrease spontaneous activity in rats and exert anti-inflammatory and neuroprotective effects. The present study explored the possible inhibitory effect of Gui Zhi on Ma Huang-induced neurotoxicity in rats when the two herbs were used in combination. All Ma Huang and Ma Huang-Gui Zhi herb pair extracts were prepared using methods of traditional Chinese medicine and were normalized based on the ephedrine content. Two-month-old male Sprague-Dawley rats (*n* = 6 rats/group) were administered Ma Huang or the Ma Huang-Gui Zhi herb pair extracts for 7 days (ephedrine = 48 mg/kg), and locomotor activity was measured. After 7 days, oxidative damage in the prefrontal cortex was measured. Gui Zhi decreased hyperactivity and sensitization produced by repeated Ma Huang administration and attenuated oxidative stress induced by Ma Huang. The results of this study demonstrate the neuroprotective potential of Gui Zhi in Ma Huang-induced hyperactivity and oxidative damage in the prefrontal cortex of rats when used in combination.

## 1. Introduction

Ma Huang Tang is a traditional Chinese herbal preparation composed of two herbs,* Herb Ephedra *(Ma Huang in Chinese) and* Ramulus Cinnamomi *(Gui Zhi in Chinese), which mutually potentiate their activities. These herbs, when combined at a crude weight ratio of 3 : 2, are used to treat asthma, nose and lung congestion, and fever with anhidrosis. These effects have also been confirmed experimentally [1, 2].

Ma Huang is derived from* Ephedra sinica *Stapf,* Ephedra intermedia *Schrenk et C. M. Mey., and* Ephedra equisetina *Bge. and contains 0.5–2.5% (by weight) total alkaloids, of which ephedrine accounts for between 30% and 90%. Ma Huang has been used to treat various respiratory conditions in China for many years, with only three related poisoning cases due to overdose reported in the literature [3–5]. Further, the Chinese Pharmacopoeia reports that Ma Huang is nontoxic suggesting that it is safe for use when applied correctly. However, several cases of Ma Huang poisoning have been reported, due to overdose and/or long-term consumption of the herb, or by using preparations containing ephedrine without the supervision of qualified healthcare professionals [6–11].

Ephedrine is an amphetamine with a pharmacological profile similar to d-amphetamine and methamphetamine. It functions as a central nervous system (CNS) stimulant; thus, ephedrine can induce insomnia, nervousness, tremors, and anxiety [12]. Long-term therapy with high doses of ephedrine may cause psychotic episodes such as paranoia, hallucinations, and other mental disturbances [13–15]. Death has been reported following chronic use of Ma Huang extract [16]. In our previous study, we found that chronic administration of Ma Huang induced obvious neurodegeneration in rat brains, with the prefrontal cortex showing the greatest effect [17].

Gui Zhi is derived from the young twigs of* Cinnamomum cassia* Presl. Its main component and major active ingredient is cinnamaldehyde, which is known for its sedative, antioxidant, and antineuroinflammation activity [18–22]. Several other active components in Gui Zhi extract also have been reported to have the neuroprotective effect [23, 24]. As the compounds in Gui Zhi extract show a sedative and anti-inflammatory effect, we hypothesized that Gui Zhi may exhibit a similar neuroprotective effect against Ma Huang-induced hyperactivity and CNS oxidative damage in the rat prefrontal cortex when used together.

## 2. Materials and Methods

### 2.1. Chemicals

(−)-Ephedrine hydrochloride, acetonitrile (HPLC grade), potassium dihydrogen phosphate, and triethylamine (reagent grade) were from Sigma (St. Louis, MO, USA).

### 2.2. Preparation of the Crude Extracts from Ma Huang and the Ma Huang-Gui Zhi Herb Pair

Ma Huang (*Herb Ephedra*, the dried stems of* Ephedra sinica *Stapf) and Gui Zhi (*Ramulus Cinnamomi*, the dried twigs of* Cinnamomum cassia* Presl), originating from inner Mongolia and the Guangxi Province of China, respectively, were supplied by Guangzhou Zhixin Pharmaceutical Co., Ltd. (Guangzhou, China) in November 2013. The identity of the herbs was verified by Professor Ma Ji (School of Traditional Chinese Medicine, Southern Medical University, Guangzhou, China).

Ma Huang was soaked in double distilled water (10 times the weight) for 30 min and then refluxed for 50 min. The extract was filtered through filter paper to remove the insoluble materials. After filtration, the extract was concentrated by rotary evaporation at 45°C and adjusted to a final volume containing the required concentration of the extract. The extract was named E and stored at 4°C until use.

The Ma Huang-Gui Zhi herb pair was prepared with crude weight ratios of 3 : 1, 3 : 2, and 3 : 4. Ma Huang was soaked in double-distilled water (10 times the weight) for 30 min and then refluxed for 20 min. Gui Zhi was then added, and the herb pair was refluxed for an additional 30 min. The extracts were filtered through filter paper to remove the insoluble materials. After filtration, the extracts were concentrated by rotary evaporation at 45°C and adjusted to a final volume containing the required concentration. The extracts were named W1 (3 : 1), W2 (3 : 2), and W3 (3 : 4) and stored at 4°C. When used, all frozen extracts were thawed until they reached room temperature.

### 2.3. HPLC Analysis

The HPLC instrument used was an Agilent HPLC Model 1100 (Palo Alto, CA, USA) equipped with an Agilent 1100 vacuum degasser, quaternary pump, autosampler, column compartment, and diode-array detector. Separation was performed on an Alltima Phenyl Column (250 × 4.6 mm i.d., 5 *μ*m particle size). The HPLC column was equilibrated with a mobile phase containing acetonitrile-water-potassium dihydrogen phosphate-triethylamine (40 mL : 960 mL : 2.72 g : 2.8 mL), at pH 3.43. The wavelength of the UV detector was set at 210 nm. After filtering with 0.45 *μ*m nylon filters, 10 *μ*L of each extract was injected into the HPLC system. The column temperature was held at 25°C and the isocratic flow rate was maintained at 0.6 mL/min.

### 2.4. Animals and Dose Formulation

Male Sprague-Dawley rats weighing 180–220 g were obtained from the Southern Medical University Experimental Animal Center (Guangzhu, China). All animals were provided with food and water* ad libitum* and allowed to adapt to the experimental conditions (temperature, 21°C ± 2°C; humidity, 50–60%) for 1 week. The studies were carried out according to the Guide for the Ethical Care and Use of Laboratory Animals, and approval for the animal studies was obtained from the Ethical Committees of Southern Medical University.

A vehicle solution was prepared using sterile distilled water and 0.9% (w/v) sodium chloride. The Ma Huang and Ma Huang-Gui Zhi preparations were adjusted based on their ephedrine content, such that the levels of Ma Huang in each extract were normalized by the ephedrine content. All animals (6 animals per treatment group) received a single oral gavage dose of the stock solutions at a volume of 10 mL/kg body weight.

### 2.5. Behavioral Assessment

The standard open field test is commonly used to assess locomotor, exploratory, and anxiety-like behavior in laboratory animals. The distance traveled (in meters) was the primary measurement collected, consistent with published studies on ephedrine-induced hyperactivity [25].

The open field used in this experiment consisted of an 80 × 80 cm closed plastic apparatus with 40 cm-high walls. The apparatus was kept in a room under dim light and the tests were undertaken during the light-on phases of the cycle, between 9 a.m. and 1 p.m. On each of the 7 days, animals were transported to the testing room and placed into the acrylic cage. After 20 min, two animals were tested simultaneously and administered a single oral gavage dose of the stock solutions. One hour after administration, the session was started by placing the rats in the central area of the open field. Each session lasted 5 min. The movements of the rats were tracked with two digital cameras, and the movies were analyzed by the computer software Smart (RWD Life Science, China). The number of total and central squares crossed and the frequencies of rearing were measured on the first day [26]. The area surrounding the open field was defined as zone 1, while the subcentral area and central area were defined as zone 2 and zone 3, respectively. The placement of only one, two, or three paws in a square followed by a return to the previous square was not considered as a crossing. In order to eliminate any olfactory cues, the apparatus was cleaned with mild detergent after each individual test.

### 2.6. Biochemical Assessment

Biochemical tests were conducted after performing the last behavioral task. The animals were sacrificed by decapitation. The brains were removed and rinsed with ice cold isotonic saline. The prefrontal cortex was separated from the brain on a stainless steel plate on ice, and the samples were homogenized with ice-cold 0.1 mmol/L phosphate buffer (pH 7.4). The homogenates (10% w/v) were centrifuged at 10,000 ×g for 15 min, and the supernatants were used for the biochemical analysis.

The total activity of superoxide dismutase (SOD), catalase (CAT), and glutathione peroxidase (GSH-Px) and the malonic dialdehyde (MDA) and nitric oxide (NO) content in the supernatants were measured using commercial kits (Nanjing Jiancheng Bioengineering Inst., China) on a microplate reader (Thermo Fisher Scientific, Inc., USA). The results of the SOD and GSH-Px analysis were expressed as U/*μ*g of tissue (wet weight), while the CAT results were expressed as U/g. The results of MDA and NO analysis were expressed as nmol/*μ*g of tissue (wet weight).

### 2.7. Statistical Analysis

Values were expressed as the mean ± SEM. The behavioral assessment data were analyzed by repeated measures two-way analysis of variance (ANOVA). The biochemical estimations were separately analyzed by one-way ANOVA. Post hoc comparisons between groups were made using Tukey's test. *P* values less than 0.05 (*P* < 0.05) were considered significant.

## 3. Results

### 3.1. Ephedrine in the Crude Extracts of Ma Huang and Ma Huang-Gui Zhi

**Figure 1:**
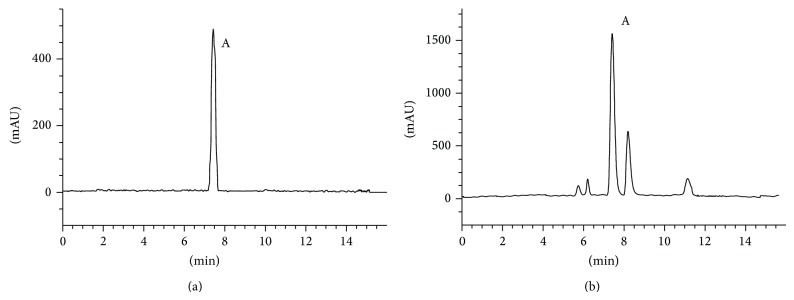
The HPLC profiles of ephedrine in standard solution (a) and typical extracts containing Ma Huang (b). A: (−)-ephedrine.

**Figure 2:**
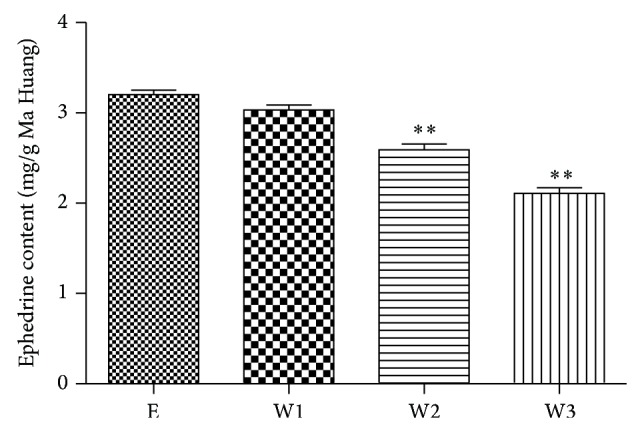
The ephedrine content of the Ma Huang and Ma Huang-Gui Zhi herb pair extracts. The values are the means ± SEM obtained from three replicate injections. ^*^ *P* < 0.05, ^**^ *P* < 0.01 as compared to E.

The HPLC profiles of ephedrine in a standard solution (a) and typical extracts containing Ma Huang (b) are shown in Figure 1. After substitution of the peak areas in the corresponding regression equations of the ephedrine standard curve, the ephedrine contents of E, W1, W2, and W3 were quantified (Figure 2). The content of ephedrine in E was the highest among the four preparations. Increasing the proportion of Gui Zhi in the Ma Huang-Gui Zhi herb pair significantly decreased the ephedrine content. These results indicated that the dissolution of ephedrine was affected when Ma Hung and Gui Zhi were prepared together.  Table 1 shows the nomenclature for the Ma Huang or Ma Huang-Gui Zhi herb pair extracts, as well as the adjusted concentrations.

### 3.2. The Effects of Acute Treatment with Ma Huang and Ma Huang-Gui Zhi on Rat Behavior

In the open field test, all of the rats administered Ma Huang or the Ma Huang-Gui Zhi herb pair exhibited higher total locomotor activity than the saline treated rats (Table 2). However, the hyperactivity induced by Ma Huang was inhibited by Gui Zhi in a dose-dependent manner. Examination of anxiety parameters (Table 2) showed that E decreased central locomotion. However, W2 and W3 increased the time spent in the central area compared to rats treated with E. Vertical exploration was reduced in E-, W1-, and W2-treated rats, compared to the saline group (Table 2). All rats treated with W1, W2, and W3 exhibited an increased rearing frequency compared to E-treated rats.

### 3.3. The Effects of Repeated Administration of Ma Huang and Ma Huang-Gui Zhi on Locomotor Activity

**Figure 3:**
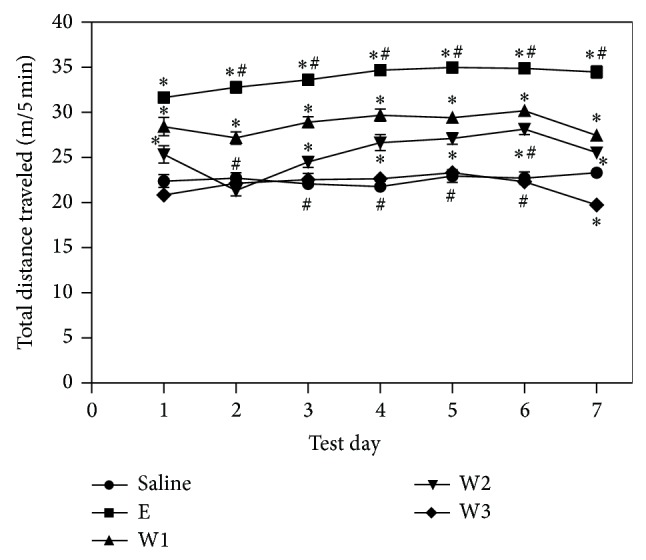
The effects of repeated Ma Huang and Ma Huang-Gui Zhi administration on locomotor activity. The data represents the mean ± SEM of six rats. ^*^ *P* < 0.05 indicates a significant difference compared to the saline treated group at each time point. ^#^ *P* < 0.05 indicates a significant difference compared to the corresponding group on day 1.

Rats were administered saline, E, W1, W2, and W3 daily for 7 consecutive days to determine the effect of repeated drug administration on locomotor activity (Figure 3). Analysis revealed that the dose group had a significant effect [*F*(4, 25) = 828.36, *P* < 0.01] on the total distance traveled, and analysis of the time course revealed that the test day also had a significant effect [*F*(6, 150) = 10.89, *P* < 0.01]. A dose group × test day interaction was also observed [*F*(24, 150) = 3.28, *P* < 0.01]. There was no significant difference in the locomotor activity of rats administered saline and W1 over the 7-day test. Rats administered E [*F*(6, 35) = 4.24, *P* < 0.01] displayed greater locomotor activity on days 2–7 than on day 1. Further, locomotor activity was greater on days 3–7 in rats administered W2 [*F*(6, 35) = 10.22, *P* < 0.01] than in rats administered saline. The rats treated with W3 [*F*(6, 35) = 6.52, *P* < 0.01] showed no significant difference in locomotor activity from rats administered saline on days 2–6. For all three herb pair groups, locomotor activity was significantly decreased on day 7 as compared to day 6. The rats treated with E showed behavioral sensitization. Groups treated with W1, W2, and W3 did not show behavioral sensitization as ephedrine.

### 3.4. Effects of Ma Huang and Ma Huang-Gui Zhi on Oxidative Stress in the Rat Prefrontal Cortex

Chronic administration of E significantly increased the levels of MDA and NO in the prefrontal cortex compared to the saline group (*P* < 0.01). W1, W2, and W3 treatment reduced MDA and NO levels as compared to E-treated rats (*P* < 0.01). This finding suggested that Gui Zhi decreased MDA and NO production induced by Ma Huang, therefore indicating that Gui Zhi reduces lipid peroxidation caused by Ma Huang.

**Figure 4:**
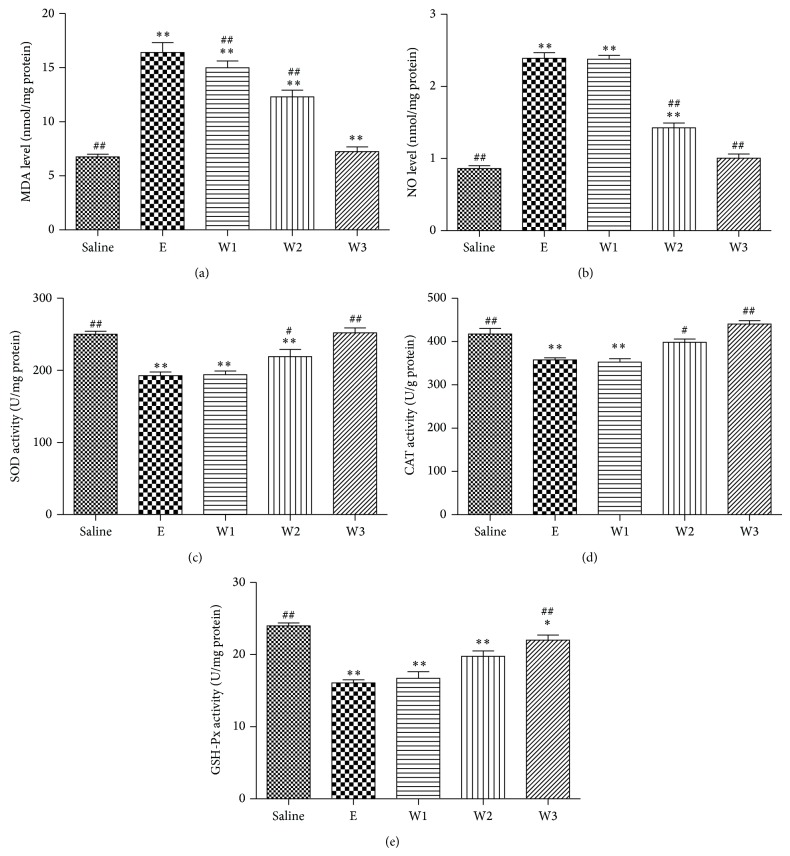
Each bar represents the mean ± SEM of six rats. ^*^ *P* < 0.05, ^**^ *P* < 0.01 indicates a significant difference compared to the saline treated group; ^#^ *P* < 0.05, ^##^ *P* < 0.01 indicates a significant difference compared to E-treated rats (ANOVA).

The total SOD, CAT, and GSH-Px activity in the prefrontal cortex of E-treated rats was significantly lower than that of the saline group (*P* < 0.01) (Figure 4). However, Gui Zhi counteracted the effects of Ma Huang on SOD, CAT, and GSH-Px in the W1-, W2-, and W3-treated groups. In rats treated with W3, SOD, and CAT activities remained at normal levels.

Chronic administration of W1, W2, and W3 in rats significantly attenuated oxidative damage, as indicated by reductions in MDA and NO concentration, and decreased SOD, CAT, and GSH-Px activities, as compared to the E-treated rats.

## 4. Discussion

In this study, we demonstrated for first time that Gui Zhi has a neuroprotective effect against Ma Huang-induced hyperactivity and CNS oxidative damage in the rat prefrontal cortex when used in combination.

Multiherb therapy is an essential component of traditional Chinese medicine. Herb pairs, a unique combination of two herbs in the clinic, is the most fundamental and the simplest form of multiherb therapy used to achieve a specific efficacy [27]. Many herb pairs were recorded in the* Treatise on Febrile and Miscellaneous Diseases* and in the* Synopsis of the Golden Chamber*. There are several methods to determine herb compatibility, including singular application, mutual promotion, mutual assistance, mutual restraint, mutual detoxification, mutual inhibition, and mutual intoxication, which are called the “seven relations of Chinese Medicine.” However, herb pairs used for mutual potentiation are the most common according to Chinese records and classic Chinese books about herbs. Mutual potentiation, also called as mutual promotion, significantly improved pharmacological efficacy compared to individual herbs [28]. The Ma Huang-Gui Zhi herb pair is a classic herb pair used for mutual potentiation, and most previous studies have focused on mutual promotion. Due to the adverse effects of Ma Huang, its medicinal use has been restricted. Further, it can only be prescribed by registered traditional Chinese medicine practitioners. However, the potential mutual detoxification effect of the herb pair has not been thoroughly considered and may be the reason why Ma Huang is a relatively safe medicinal herb in clinical practice.

Due to the complexity of chemical compositions in traditional Chinese medicine, most herb pair studies normalize the dosage according to the amount of crude drug. In the present study, we found that ephedrine levels in the Ma Huang-Gui Zhi herb pair were significantly decreased, indicating that the main component is affected when the herb pair is boiled together. Therefore, it was essential to adjust the preparations according to the ephedrine content. Further, chemical analyses of ephedrine in Ma Huang typically use ethanol as a solvent, which yields a greater amount of ephedrine. The solubility of ephedrine in water is 50 g/L, whereas it is completely soluble in ethanol. Therefore, if our Ma Huang samples were extracted with other organic solvents, the yields of ephedrine may have been higher. However, in this study, Ma Huang was extracted with water to simulate the use of the herb in traditional Chinese medicine.

Previous work has shown that the open field test can be used to measure rodent locomotion and emotional state. In the open field test, crossing and rearing behaviors are commonly measured to study the effects of central excitatory drugs, and other classes of drugs, on rodent behavior in a new environment [29]. Studies have demonstrated that, in an unfamiliar environment, the animal tends to ambulate on the periphery of the box where the walls provide security, a behavior called thigmotaxis. In contrast, animals treated with central excitatory drugs tend to display decreased central area exploration and an increase in total distance traveled [30]. All of the herbal preparations containing Ma Huang stimulated animal ambulation. Further, Ma Huang-treated rats exhibited a higher total locomotor activity compared to the herb pair treated groups. Gui Zhi produced mild sedation, observed by a reduced total locomotion and increase in rearing, central crossing, and time spent in the central area of the apparatus, in a dose dependent manner.

In rodents, acute ephedrine injection produces hyperactivity [31] and repeated injection results in sensitization to ephedrine-induced hyperactivity [25]. Conversely, plants containing cinnamaldehyde have sedative effects in animal models. The effects of cinnamaldehyde on general behavior, locomotor activity, and body temperature have been studied in mice. The results indicate that 30 mg/kg cinnamaldehyde had no effect; however, doses of 60 mg/kg and above showed decreased spontaneous activity in mice. At doses of 125 mg/kg and higher, locomotor activity induced by apomorphine or methamphetamine was suppressed by cinnamaldehyde [32]. In the present study, we found that the effect of Ma Huang is similar to ephedrine. Acute administration of Ma Huang produced hyperactivity, and, with repeated drug administration, sensitization developed, consistent with published research [33]. We demonstrated for first time that Gui Zhi inhibited Ma Huang-induced hyperactivity in the CNS. Gui Zhi also altered Ma Huang-induced hyperactivity after acute administration of the Ma Huang-Gui Zhi herb pair and prevented the development of locomotor sensitization to Ma Huang.

In addition to behavioral evaluations, this study also showed that Gui Zhi prevented Ma Huang-induced central nervous system oxidative injury* in vivo*, through the reduction of NO and MDA levels and a concomitant increase in SOD, CAT, and GSH-Px activity in the prefrontal cortex. Central nervous system oxygen toxicity (CNS-OT), which is characterized by tonic–clonic convulsions and sudden loss of consciousness, may be fatal if it occurs while diving [34]. Signs of CNS-OT include nausea, dizziness, muscle twitching, and visual disorders. These disruptions may progress to neuronal damage and death in some severe cases [35]. Although the precise mechanism of CNS-OT remains poorly understood, it is accepted that MDA and NO, as well as damaged antioxidant defenses, may mediate the hyperoxic insults [36, 37]. NO levels can be increased when SOD reduces O_2_^−^ levels, as O_2_^−^ is the substrate for SOD that inactivates NO in hyperoxia [38]. As a result, SOD seems to mediate CNS-OT because of its contribution to NO enhancement. Mice that overexpress SOD are likely to have more severe CNS-OT. Conversely, inhibiting SOD can prolong the latency to hyperoxic seizures in animals [39, 40]. In contrast, CAT was shown to have a protective effect against CNS-OT [41, 42]. Antioxidants may be activated by mild HBO_2_ exposure due to oxidative stress [43, 44] but much of their activity may be consumed by the dramatic increase in ROS during extreme exposure, as in the present study, leading to an imbalance between ROS production and clearance [45].

Ephedrine is similar in several respects to d-amphetamine and methamphetamine. Multiple doses of ephedrine can cause severe hyperthermia and neurodegeneration in the rat brain [46]. It was reported that chronic administration of ephedrine induced obvious neurodegeneration in mice prefrontal cortex [47, 48]. In our previous study, we also found that chronic administration of Ma Huang induced obvious neurodegeneration in rat brains, with the prefrontal cortex showing the greatest effect [17]. The neurodegeneration in prefrontal cortex may be the result of excitotoxic mechanisms, followed by obvious inflammation and CNS-OT. It was reported that cinnamon has potent antioxidant activity and can scavenge ROS, including NO, the superoxide anion, and peroxynitrite [49]. Additionally, cinnamon extract can suppress NO production and iNOS expression in LPS-activated RAW264.7 macrophages with a relatively low IC_50_ [50]. Indeed, cinnamaldehyde potently inhibits macrophage-mediated inflammation. Recently, cinnamaldehyde has been shown to mitigate the inflammatory response and elevate antioxidant enzyme activities in a carrageenan-induced edema animal model [51]. Furthermore, the mechanism underlying cinnamaldehyde-mediated downregulation of lipopolysaccharide- (LPS-) induced cytokine expression in several peripheral macrophages has been extensively investigated. Cinnamaldehyde does not interfere with LPS binding to cell membrane receptors, specifically toll-like receptor 4 (TLR4) but can suppress TLR4 oligomerization and attenuate the LPS-elicited intracellular signaling process [19, 52]. Cinnamaldehyde reduces LPS-induced intracellular ROS formation and, therefore, restores redox balance and attenuates oxidative stress-triggered signal transduction pathways, including NF-*κ*B-inducing kinase/I*κ*B*α* kinase (NIK/IKK), extracellular signal-regulated kinases (ERK), and p38 mitogen-activated protein kinases (MAPK), to inhibit activation of NF-*κ*B [22].

Several mechanisms may be involved in the protective effects of Gui Zhi on CNS-OT, including eliminating or decreasing ^•^OH, O_2_^−^ and H_2_O_2_ [53], inhibiting ^•^OH-dependent or -independent lipid peroxidation [54], restoring the activity of SOD, CAT, and GSH-Px [55–57], or inhibiting lipoxygenase, which may affect cerebrovascular tone and vascular control [58, 59]. Nevertheless, in the present study, we could not to distinguish whether Gui Zhi enhanced these antioxidases directly or indirectly via scavenging ROS. The present study confirms that chronic administration of Ma Huang increased the levels of oxidative biomarkers, which was demonstrated by elevated levels of MDA and NO and concomitant reductions in SOD, CAT, and GSH-Px activity in the prefrontal cortex. Conversely, Gui Zhi effectively protected against CNS-OT and reversed the changes in the above measures.

## 5. Conclusion

In conclusion, CNS-OT induced by Ma Huang may have resulted in part from excitotoxic mechanisms involving the indirect pathways of the prefrontal cortex and related areas. Gui Zhi displayed a neuroprotective effect against Ma Huang-induced hyperactivity and CNS-OT. The present study indicates the advantage of the two herbs used in combination. It may be the reason why Ma Huang is relative save for use in traditional Chinese medicine. Although the correlation of these findings in humans remains to be assessed, we believe our findings that the adverse effects of Ma Huang treatment reversed by Gui Zhi will have important clinical effects.

## Figures and Tables

**Table 1:** Ma Huang or Ma Huang-Gui Zhi herb pair extracts and the adjusted concentration.

Sample	Composition	Ephedrine (mg/mL)	Ephedra (g/mL)
E	Ma Huang	4.8	1.50
W1	3 : 1 (Ma Huang-Gui Zhi)	4.8	1.58
W2	3 : 2 (Ma Huang-Gui Zhi)	4.8	1.85
W3	3 : 4 (Ma Huang-Gui Zhi)	4.8	2.32

**Table 2:** Effects of acute intragastric administration in the OF test.

Group	Zone	Time (s)	P. time (%)	Crossing	Rearing	Distance traveled (m)
Saline	1	245.40 ± 17.11^#^	81.80 ± 5.70^#^	15.50 ± 1.87^#^	23.17 ± 6.31^#^	22.39 ± 1.74^#^
	2	43.77 ± 24.11	14.59 ± 8.04			
	3	5.40 ± 3.84	1.80 ± 1.20			

E	1	284.62 ± 4.62^*^	94.87 ± 1.54^*^	11.00 ± 3.58^*^	8.00 ± 3.46^*^	31.66 ± 1.48^*^
	2	14.98 ± 4.54	4.99 ± 1.51			
	3	0.23 ± 0.48	0.08 ± 0.16			

W1	1	289.23 ± 8.47^*^	96.41 ± 2.82^*^	12.83 ± 2.48	13.50 ± 4.51^∗#^	28.42 ± 2.49^∗#^
	2	8.63 ± 6.02	2.88 ± 2.01			
	3	1.97 ± 2.76	0.66 ± 0.92			

W2	1	272.92 ± 3.95^#^	90.97 ± 1.32^#^	14.33 ± 2.42^#^	17.00 ± 3.03^∗#^	25.34 ± 2.37^∗#^
	2	17.85 ± 3.17	5.95 ± 1.06			
	3	3.47 ± 3.18	1.16 ± 1.06			

W3	1	250.75 ± 15.22^#^	83.58 ± 5.07^#^	15.17 ± 1.94^#^	20.33 ± 1.75^#^	20.85 ± 0.93^#^
	2	43.02 ± 16.23^#^	14.34 ± 5.41^#^			
	3	4.30 ± 2.05^#^	1.43 ± 0.69^#^			

^*^ *P* < 0.05 indicates a significant difference compared to the saline treated group; ^#^ *P* < 0.05 indicates a significant difference compared to the E-treated group.
